# Rapid changes in major nutrients and fungal community during early decomposition of *Betula platyphylla* litter on volcanic lava plateau

**DOI:** 10.3389/ffunb.2026.1773867

**Published:** 2026-03-13

**Authors:** Yan Wei, Jiahui Cheng, Yongwu Liu, Tianzi Xia, Qingyang Huang, Fan Yang, Ruifeng Fan

**Affiliations:** 1 School of Pharmacy, Heilongjiang University of Chinese Medicine, Harbin, Heilongjiang, China; 2 Institute of Natural Resources and Ecology, Heilongjiang Academy of Sciences, Harbin, Heilongjiang, China; 3 Centre of Non-Clinical Safety Assessment, Heilongjiang University of Chinese Medicine, Harbin, Heilongjiang, China

**Keywords:** *Betula platyphylla*, function prediction, fungal community, litter decomposition, nutrients, volcanic lava

## Abstract

Volcanic eruptions deposit lava that covers the original soil and alters biogeochemical cycles, leading to the formation of unique ecological characteristics. Litter decomposition is an important part of the nutrient cycling in volcanic forest ecosystems. This study aimed to investigate the dynamic coupling between litter nutrient release and fungal community succession during the decomposition of pioneer *Betula platyphylla* in a volcanic forest. We conducted an *in situ* decomposition experiment combined with Illumina MiSeq high-throughput sequencing and fungal functional prediction. We found that, on the lava plateau, after 18 months of decomposition, *Betula platyphylla* litter remains in the early stages of decomposition, with rapid changes occurring in its nutrient composition and fungal community structure. (*p* < 0.05). The diversity and composition of fungal communities in *Betula platyphylla* litter differed significantly among the four sampling periods. The succession of fungal communities, dominated by Ascomycota and Basidiomycota, was primarily driven by changes in litter mass remaining and key nutrient concentrations (C, N and P). Our results offer valuable insights for further investigations into the dynamics of fungal communities during litter decomposition in volcanic forest ecosystems.

## 1 Introduction

Forest litter, produced during plant life cycles, serves as a crucial reservoir of organic matter and nutrients for both plants and microorganisms (Lladó et al., 2017). Its decomposition is a key driver of nutrient cycling, plant growth, biodiversity, and terrestrial carbon balance (Leppert et al., 2017). Microorganisms colonizing the litter surface primarily initiate and regulate this decomposition process (Cline and Zak, 2015), through their uptake and release of elements (Drake et al., 2011). Fungi are particularly integral to litter decomposition. As decomposition progresses, the diversity and biomass of these litter-associated fungal communities typically increase, with different fungal taxa showing stage-specific dynamics (Sun et al., 2021; Zhao et al., 2021; Liu et al., 2022). Furthermore, the composition and diversity of litter fungi are significantly influenced by tree species (Zhao et al., 2023). Consequently, understanding fungal community composition and diversity has attracted growing research interest (Floudas et al., 2020; Majchrowska-Safaryan and Tkaczuk, 2021; Contos et al., 2024). Among the successive stages of leaf litter decomposition, the early decomposition phase holds particular ecological significance. This decomposition phase accounts for approximately 40% of mass loss, during which litter undergoes fragmentation, physical leaching, and organic metabolism (Heim and Frey, 2004). These processes degrade soluble compounds, non-cellulose, non-hemicellulose, and non-lignin components, while pioneer microbial communities within the litter begin breaking down refractory structural components (Bray et al., 2012; Hu et al., 2017).

Volcanic eruptions, a prevalent form of ecological and geological disturbance, create distinctive ecosystems that serve as valuable models for investigating biodiversity responses to novel habitat formation (Elser et al., 2015). Extensive research has focused on the impacts of eruptions on plants and soils (Zhang et al., 2024). For example, in Latin America, soils derived from young volcanic ash exhibit reduced fungal abundance. This reduction directly impairs the conversion of soil organic nutrients into plant-available forms, thereby limiting plant nutrient uptake (Joergensen and Castillo, 2001). The eruption of the Mutnovsky volcano has been associated with increased diatom diversity in nearby terrestrial ecosystems (Fazlutdinova et al., 2021). Furthermore, volcanic soils can alter soil microbial community structure, increase the relative abundance of beneficial bacteria, and improve the micro-ecological environment (Xue et al., 2024). Recent research on the vegetation ecology of the Wudalianchi volcanic region has expanded significantly, primarily investigating dominant plant adaptability, soil microbial diversity and biomass, and vegetation-soil interactions (Huang et al., 2020; Xie et al., 2022; Yin et al., 2022).

*Betula platyphylla* thrives in harsh environments characterized by cold temperatures, low soil fertility, and aridity. This makes it a key pioneer species in primary succession following volcanic eruptions and a prevalent species on lava plateaus. Compared to coniferous species, broad-leaved trees such as *Betula platyphylla* exhibit a higher litter decomposition rate (Lu et al., 2019). This rapid decomposition releases essential nutrients, facilitating nutrient cycling that is crucial for forest vegetation recovery in volcanic ecosystems. Consequently, *Betula platyphylla* plays a vital role in vegetation establishment, succession, and ecosystem nutrient cycling in volcanic forests (Xie et al., 2023). Volcanic lava soils are characterized by nutrient deficiency, loose texture, low water and nutrient retention capacities, and a distinct microbial community composition, differing from typical forest, grassland, or farmland soils (Xie et al., 2021). However, the characteristics of litter decomposition in this specific environment remain poorly understood. We hypothesize that: (1) In volcanic lava habitats, even after 18 months of decomposition, *Betula platyphylla* leaf litter remains in the early stages of decomposition, with significant changes occurring in litter major nutrient and structural components. (2) As litter decomposition progresses, these pronounced nutrient shifts drive the succession of fungal communities and the transformation of fungal functional traits. To validate these hypotheses, we conducted an *in-situ* litter decomposition experiment for *Betula platyphylla* in the volcanic lava and explored the dynamic coupling relationship between litter nutrient release and fungal community succession.

## 2 Materials and methods

### 2.1 Overview of the study area

This study was conducted at the Heihe (Wudalianchi) National Forest Ecosystem Research Station (48°42′ N, 126°07′ E), located in the Wudalianchi area of northwestern Heilongjiang Province, China. The region experiences a cold, humid temperate continental monsoon climate, with a mean annual temperature of -0.5 °C, annual precipitation of 476 mm, and a frost-free period of 121 days. The volcanic plateaus of Wudalianchi were formed by historic lava flows and volcanic ash deposits, which have given rise to infertile but well-drained soils.

### 2.2 Experimental design

The litter of *Betula platyphylla*, the dominant tree species on the Wudalianchi volcanic lava plateau, was collected in late September 2021. The litters were blended evenly at room temperature and dried to a constant weight. Subsequently, 10.0 g samples were weighed and packed into a litter bag of 100 mesh, 35 cm × 25 cm in size. Six separate and flat plots were chosen for the decomposition study within the research area, with each site covering an area of 50 m^2^. In mid-October 2021, the soil surface was cleared of existing litter and debris. The litter bags were then evenly distributed across the plots with a minimum spacing of 5 cm between bags, positioned to simulate natural litterfall, and secured using plastic ground nails (Huang et al., 2023).

### 2.3 Sample collection

**Figure 1:**
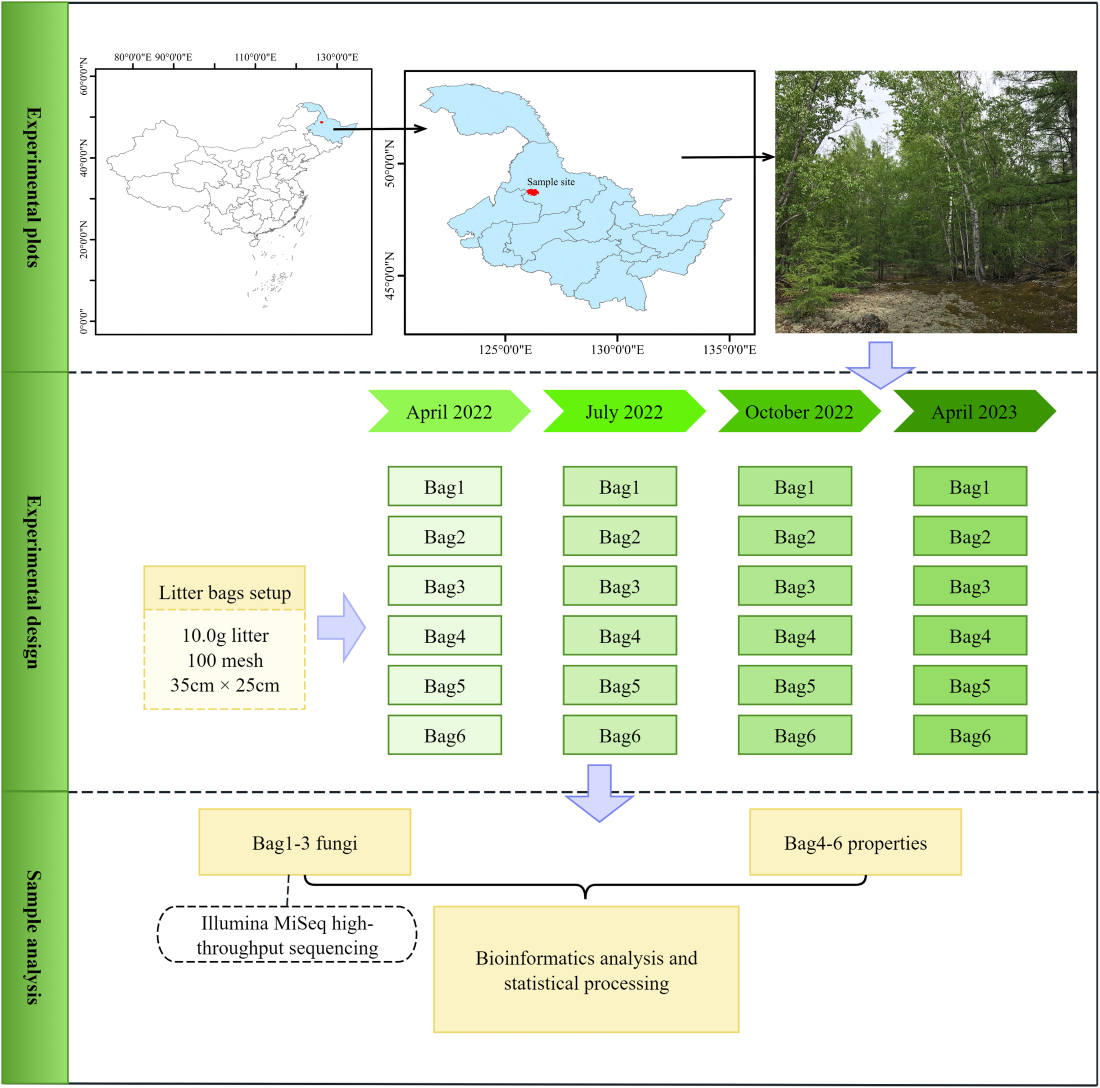
The setup of the litter decomposition experiment.

The experiment involved four sampling sessions in April 2022 (t1), July 2022 (t2), October 2022 (t3), and April 2023 (t4). At each sampling time, one bag was retrieved from every treatment plot. From the six bags collected per sampling, three were processed to determine mass loss. These bags were sterilized at 105 °C to deactivate enzymes and then oven-dried at 80 °C to constant weight for the calculation of litter mass remaining. The dried material was subsequently ground, sieved, and analyzed for chemical composition. Additionally, three replicate samples were frozen in liquid nitrogen for fungal content analysis in the litter (Huang et al., 2023) (**Figure 1**). DNA extraction and fungal high-throughput sequencing were outsourced to Shanghai Major Biomedical Technology Co., Ltd. (Shanghai, China).

### 2.4 Analysis of litter properties

The combined amounts of total nitrogen (N) and organic carbon (C) in the litter were determined using an elemental analyzer (EA300, Euro Vector, Foggia, Italy) after drying and grinding pretreatment. Total phosphorus (P) concentration was measured by the molybdenum-antimony colorimetric method following digestion with concentrated sulfuric acid and hydrogen peroxide (Han et al., 2022; Li et al., 2023). The structural components (lignin, cellulose, and hemicellulose) were quantified colorimetrically using a microplate reader (Multiskan Sky, Thermo Scientific, USA). Specifically, lignin concentration was measured based on the ultraviolet absorption value of its acetylation product at 280 nm, with alkaline lignin serving as the standard. Cellulose concentration was determined at 620 nm through the color reaction between its acid hydrolysis product, glucose, and anthracone-sulfuric acid reagent. The hemicellulose concentration was quantified via the color reaction between the reducing sugars produced from its acid hydrolysis and the DNS reagent at 540 nm (Osono and Takeda, 2005). The above three colorimetric determinations were conducted in triplicate, and standard curves were generated. The results were expressed as dry weight concentration (g·kg^-1^).

### 2.5 DNA extraction, PCR amplification, and Illumina MiSeq sequencing

Total genomic DNA was extracted from litter samples using the E.Z.N.A.^®^ Power Soil DNA Kit (Omega Bio-tek, Norcross, GA, USA). DNA concentration and purity were measured with a NanoDrop 2000 spectrophotometer (Thermo Fisher Scientific, Wilmington, DE, USA), and integrity was verified by 1% agarose gel electrophoresis. Qualified DNA was amplified with primers 2 ITS1F (5’-CTTGGTCATTTAGAGGAAGTAA-3’) and ITS2 (5’-GCTGCGTTCTTCATCGATGC-3’). PCR amplification was performed under the following conditions: initial denaturation at 95 °C for 3 min; 27 cycles of denaturation at 95 °C for 30 s, annealing at 55 °C for 30 s, and extension at 72 °C for 45 s; followed by a final extension at 72 °C for 10 min. The PCR products were checked on a 2% agarose gel, purified using the AxyPrep DNA Gel Extraction Kit (Axygen Biosciences, Union City, CA, USA), and quantified with the QuantiFluor™-ST system (Promega, Madison, WI, USA). Purified amplicons were subjected to paired-end sequencing on an Illumina MiSeq platform by Shanghai Major Biomedical Technology Co., Ltd. (Shanghai, China).

### 2.6 Bioinformatics analysis and statistical processing

Raw paired-end reads were processed using FASTP (v0.19.6) for quality control and adapter trimming (Chen et al., 2018). Forward and reverse reads were then merged using FLASH (v1.2.11) (Magoč and Salzberg, 2011). The merged sequences were filtered to remove low-quality reads, ambiguous bases, and chimeras using the UCHIME algorithm within USEARCH (v10.0) (Edgar et al., 2011). Operational taxonomic units (OTUs) were clustered at 97% similarity from the resulting high-quality sequences using the UPARSE algorithm (Edgar, 2013). Taxonomic assignment was performed against the UNITE (v8.0) fungal ITS database using the RDP Classifier (v2.13) with a confidence threshold of 0.7 (Wang et al., 2007; Kõljalg et al., 2013). To eliminate analytical bias caused by uneven sequencing depth across samples, rarefaction was applied to the OTU abundance table to normalize all samples to an identical sequencing depth of 57,591 high-quality reads using USEARCH (v10.0). Fungal community composition was analyzed at multiple taxonomic levels based on these assignments.

Alpha diversity indices, including Sobs, Ace, Pd, Chao, Shannon and Simpson, were calculated using mothur software (Schloss et al., 2009). Specifically, the Sobs index represents the observed number of fungal taxa. The Ace and Chao indices are estimators of total species richness, with the Ace index emphasizing rare species and the Chao index focusing on singletons (Chao and Yang, 1993). The Pd index quantifies phylogenetic diversity (Faith, 1992). The Shannon index reflects species diversity, while the Simpson index indicates species dominance (Shannon, 1948; Simpson, 1949). The ecological functions of fungi were predicted using the FUNGuild platform (Nguyen et al., 2016). Differences in litter mass remaining, physicochemical properties, and fungal community metrics across decomposition stages were assessed using one-way ANOVA (*p* < 0.05) in SPSS (v19.0). *Post hoc* analysis was conducted using the Least Significant Difference (LSD) test. Statistical analyses were further supported using R software (v3.5.1). Beta diversity was analyzed via non-metric multidimensional scaling (NMDS) based on Bray-Curtis distances using the vegan package. The relationship between fungal community composition and litter physicochemical variables was examined using redundancy analysis (RDA). All data are presented as mean ± standard error (SE).

## 3 Results

### 3.1 Changes in nutrient elements and structural components of litter

The litter mass remaining after 18 months of decomposition is 61.73%, indicating an early decomposition stage (**Table 1**). The concentrations of carbon (C) and phosphorus (P) in the litter were significantly higher at the earlier decomposition stages (t1 and t2) than at the later stages (t3 and t4) (*p* < 0.05). The concentration of nitrogen (N) increased significantly from t1 to t2 but decreased significantly from t3 to t4 (*p* < 0.05).

**Table 1:** Plant litter mass remaining, nutrient elements and structural components across different decomposition stages.

Sampling Date	t1	t2	t3	t4
LMR (%)	83.07 ± 0.38 ^a^	70.60 ± 0.95 ^b^	62.80 ± 0.16 ^c^	61.73 ± 0.27 ^c^
C (g kg^-1^)	506.95 ± 1.26 ^a^	503.14 ± 3.31 ^a^	494.05 ± 2.75 ^b^	453.63 ± 2.10 ^c^
N (g kg^-1^)	20.63 ± 0.10 ^b^	24.19 ± 0.43 ^a^	23.54 ± 0.20 ^a^	19.35 ± 0.20 ^c^
P (g kg^-1^)	5.10 ± 0.11 ^a^	4.87 ± 0.05 ^b^	4.50 ± 0.02 ^c^	3.86 ± 0.03 ^d^
C:N	24.57 ± 0.10 ^a^	20.81 ± 0.28 ^c^	20.99 ± 0.06 ^c^	23.45 ± 0.20 ^b^
C:P	99.43 ± 2.44 ^c^	103.35 ± 1.79 ^c^	109.84 ± 0.89 ^b^	117.44 ± 1.08 ^a^
N:P	4.05 ± 0.09 ^b^	4.97 ± 0.14 ^a^	5.23 ± 0.06 ^a^	5.01 ± 0.03 ^a^
CL (g kg^-1^)	185.97 ± 1.49 ^a^	141.91 ± 3.18 ^c^	160.84 ± 4.37 ^b^	127.63 ± 4.14 ^d^
HC (g kg^-1^)	222.39 ± 1.88 ^a^	201.53 ± 0.67 ^b^	192.16 ± 4.68 ^b^	177.24 ± 2.90 ^c^
LG (g kg^-1^)	219.14 ± 3.44 ^a^	183.84 ± 3.07 ^b^	184.86 ± 4.41 ^b^	193.21 ± 1.30 ^b^

The data in the table represent mean ± SE (standard error) (n = 3). Different letters (a, b, c) indicate significant differences (one-way ANOVA, *p* < 0.05). LMR (litter mass remaining), LG (lignin), CL (cellulose), HC (hemicellulose). t1: sampling in April 2022, t2: sampling in July 2022, t3: sampling in October 2022, t4: sampling in April 2023.

The C:P ratio showed a significant increasing trend (*p* < 0.05), rising gradually from 99.43 at t1 to 117.44 at t4. The C:N ratio decreased significantly from t1 (24.57) to t2 (20.81) and then increased significantly from t2 to t4 (23.45). The N:P ratio was significantly higher at t2 (4.97), t3 (5.23), and t4 (5.01) than at t1 (4.05), peaking at t3. The initial concentrations of cellulose (185.97 g·kg^-1^), hemicellulose (222.39 g·kg^-1^), and lignin (219.14 g·kg^-1^) at t1 were significantly higher than those at subsequent sampling times (t2 to t4) in *Betula platyphylla* litter.

### 3.2 Fungal diversity of *Betula platyphylla* litter

Fungal diversity indices for *Betula platyphylla* litter at different decomposition stages are presented in **Table 2**. The Coverage index of *Betula platyphylla* litter, sampled across four stages, exceeded 0.996, affirming the adequacy of sampling and the fidelity of sequencing results in reflecting fungal community information. The Sobs, Ace, Pd and Chao indexes of Betula platyphylla litter from t3 and t4 markedly surpassed those from t1 and t2 (*p* < 0.05). The Shannon index reached its minimum in t1, significantly lower than the other stages. The Simpson index exhibited significantly higher values in t1 and t4 compared to t2 and t3, with t1 recording the highest Simpson index (*p* < 0.05).

**Table 2:** Litter fungal diversity indices under different decomposition stages.

Index	t1	t2	t3	t4
Sobs	179.33 ± 13.97 ^c^	331.00 ± 25.18 ^b^	524.67 ± 29.20 ^a^	463.00 ± 23.03 ^a^
Shannon	2.2306 ± 0.0483 ^b^	3.0905 ± 0.2083 ^a^	3.3686 ± 0.1291 ^a^	3.0968 ± 0.0927 ^a^
Simpson	0.1811 ± 0.0094 ^a^	0.0914 ± 0.0033 ^b^	0.0984 ± 0.0038 ^b^	0.1167 ± 0.0123 ^a^
Ace	295.38 ± 32.97 ^c^	538.72 ± 33.01 ^b^	679.19 ± 10.95 ^a^	634.91 ± 27.27 ^a^
Chao	283.55 ± 9.39 ^d^	477.53 ± 3.74 ^c^	671.29 ± 17.26 ^a^	606.42 ± 18.29 ^b^
Coverage	0.9987 ± 0.0001 ^a^	0.9979 ± 0.0003 ^b^	0.9971 ± 0.0002 ^b^	0.9973 ± 0.0003 ^b^
Pd	38.02 ± 1.78 ^c^	67.39 ± 0.87 ^b^	93.36 ± 2.47 ^a^	85.85 ± 3.58 ^a^

The data in the table represent mean ± SE (standard error) (n = 3). Different letters (a, b, c) indicate significant differences (one-way ANOVA, *p* < 0.05). t1: sampling in April 2022, t2: sampling in July 2022, t3: sampling in October 2022, t4: sampling in April 2023.

**Figure 2:**
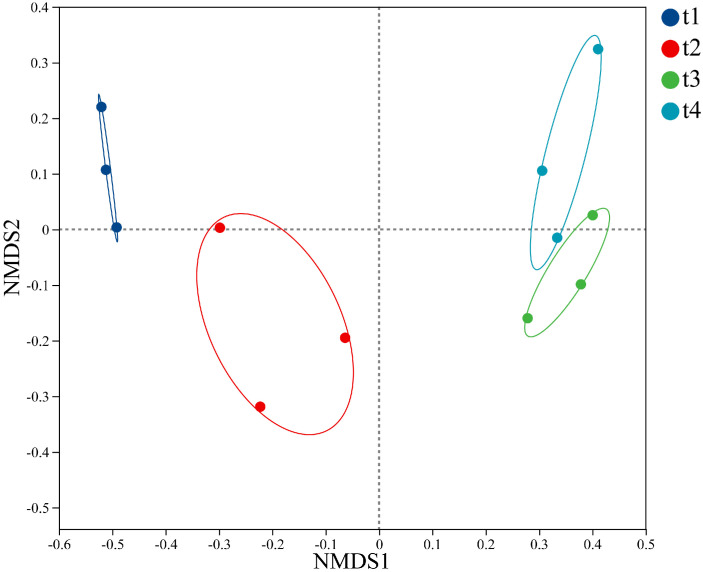
Non-metric multidimensional scaling (NMDS) analysis of different *Betula platyphylla* litter fungal communities based on bray-curtis distances. t1: sampling in April 2022, t2: sampling in July 2022, t3: sampling in October 2022, t4: sampling in April 2023.

The results of non-metric multidimensional scaling analysis of fungal communities in *Betula platyphylla* litter based on Bray-Curtis distance are shown in **Figure 2**, with a stress coefficient of 0.053. The results show that intergroup differences in the endophytic fungal communities of birch litter were significantly greater than intragroup differences across the four stages (R = 0.7994, *p* = 0.002). Clusters from stages t3 and t4 were positioned closer together, while clusters from stages t1 and t2 were positioned farther apart.

### 3.3 Fungal community composition of *Betula platyphylla* litter

**Figure 3:**
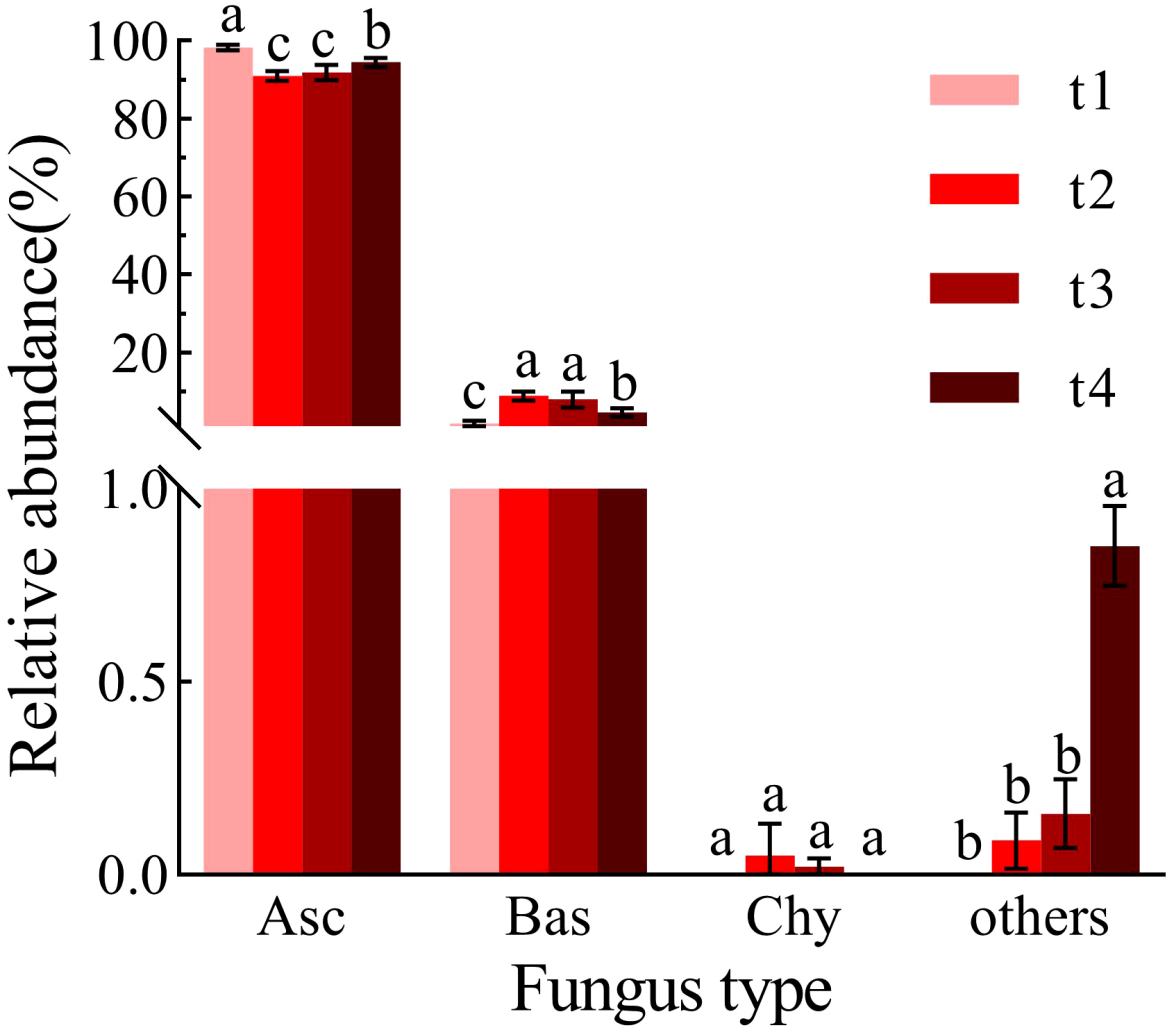
Relative abundance at the phylum level during the decomposition process. Different letters (a–c) indicate significant differences (one-way ANOVA, *p* < 0.05). Asc (Ascomycota), Bas (Basidiomycota), Chy (Chytridiomycota). t1: sampling in April 2022, t2: sampling in July 2022, t3: sampling in October 2022, t4: sampling in April 2023.

At the phylum level (**Figure 3**), the fungal community in *Betula platyphylla* litter was predominantly composed of Ascomycota, Basidiomycota, and Chytridiomycota. Among these, Ascomycota was overwhelmingly dominant, accounting for 90.99% to 98.27% of the community. Its relative abundance was highest (98.27%) at the initial sampling point (t1). Over the 18-month decomposition period, the relative abundance of Chytridiomycota remained stable, showing no significant change from t1 to t4 (*p* > 0.05). In contrast, the relative abundance of Basidiomycota was significantly lower at t1 (1.73%) than during the subsequent stages (t2 to t4) (*p* < 0.05), peaking at t2 (8.87%).

**Figure 4:**
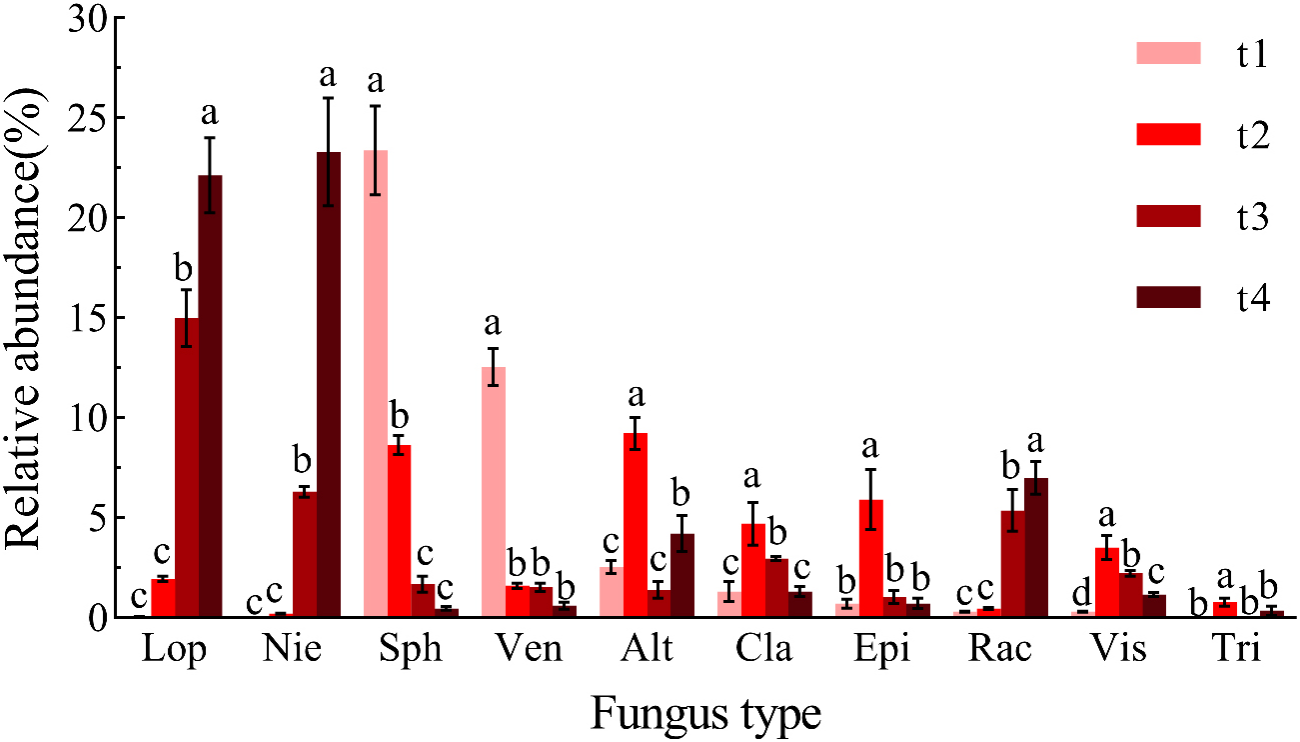
Relative abundance at the genus level during the decomposition process. Different letters **(a–c)** indicate significant differences (one-way ANOVA, *p* < 0.05). Lop (*Lophiostoma*), Nie (*Niesslia*), Sph (*Sphaerulina*), Ven (*Venturia*), Alt (*Alternaria*), Cla (*Cladosporium*), Epi (*Epicoccum*), Rac (*Rachicladosporium*), Vis (*Vishniacozyma*), Tri (*Trichothecium*). t1: sampling in April 2022, t2: sampling in July 2022, t3: sampling in October 2022, t4: sampling in April 2023.

At the genus level (**Figure 4**), the ten most abundant fungal genera in *Betula platyphylla* litter were *Lophiostoma*, *Niesslia*, *Sphaerulina*, *Venturia*, *Alternaria*, *Cladosporium*, *Epicoccum*, *Rachicladosporium*, *Vishniacozyma*, and *Trichothecium*. After 18 months of decomposition (t4), the relative abundance of *Lophiostoma* (22.11%), *Niesslia* (23.29%), and *Rachicladosporium* (6.98%) had increased significantly compared to initial levels, whereas those of *Sphaerulina* (23.37%) and *Venturia* (12.53%) had decreased significantly (*p* < 0.05). The genera *Alternaria* (9.21%), *Cladosporium* (4.68%), *Epicoccum* (5.90%), *Vishniacozyma* (3.49%), and *Trichothecium* (0.76%) were most dominant at t2, with significantly higher relative abundances than at t1, t3, or t4 (*p* < 0.05).

### 3.4 Relationship between litter nutrients, structural components and fungal community composition

**Figure 5:**
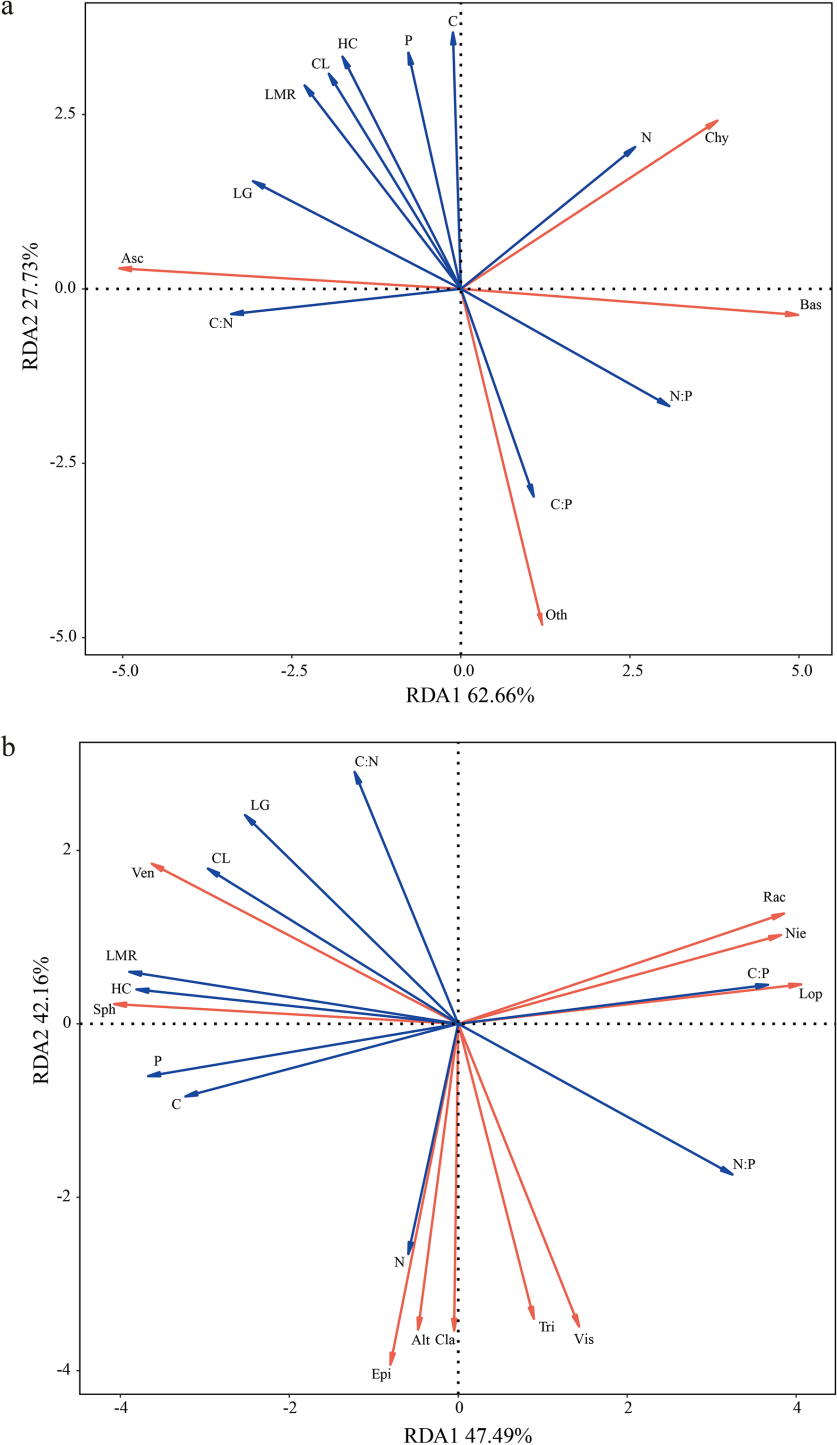
Redundancy analysis of nutrient element concentration and structural components and fungal community of litter [**(a)** phylum level, **(b)** genus level]. Red arrows represent individual fungal taxa, and blue arrows represent litter mass remaining, nutrient elements and structural components. Asc (Ascomycota), Bas (Basidiomycota), Chy (Chytridiomycota), Lop (*Lophiostoma*), Nie (*Niesslia*), Sph (*Sphaerulina*), Ven (*Venturia*), Alt (*Alternaria*), Cla (*Cladosporium*), Epi (*Epicoccum*), Rac (*Rachicladosporium*), Vis (*Vishniacozyma*), Tri (*Trichothecium*), LMR (litter mass remaining), LG (lignin), CL (cellulose), HC (hemicellulose).

The redundancy analysis (RDA) at the phylum level (**Figure 5a**) showed that the two axes together explained 90.39% of the total variance. Specifically, LMR (R^2^ = 0.872, *p* = 0.003), HC (R^2^ = 0.890, *p* = 0.001), CL (R^2^ = 0.847, *p* = 0.002), and C (R^2^ = 0.844, *p* = 0.002) showed the highest explanatory power, with LMR, HC and CL positively associated with Ascomycota and negative associations with Basidiomycota, whereas C showed a positive correlation with Chytridiomycota.

At the genus level (**Figure 5b**), the two RDA axes together explained 89.65% of the total variance. LMR (R^2^ = 0.969, *p* = 0.001), HC (R^2^ = 0.927, *p* = 0.001), CL (R^2^ = 0.790, *p* = 0.002), P (R^2^ = 0.872, *p* = 0.002) and C:P ratio (R^2^ = 0.854, *p* = 0.002) were the top explanatory variables. *Sphaerulina* and *Venturia* showed positive associations with LMR, CL, HC and P but negative associations with the C:P ratio. *Rachicladosporium*, *Niesslia*, *Trichothecium*, *Lophiostoma* and *Vishniacozyma* exhibited negative correlations with LMR, CL, HC and P while displaying positive correlations with the C:P ratio.

### 3.5 Fungal ecological function prediction of *Betula platyphylla* litter

**Figure 6:**
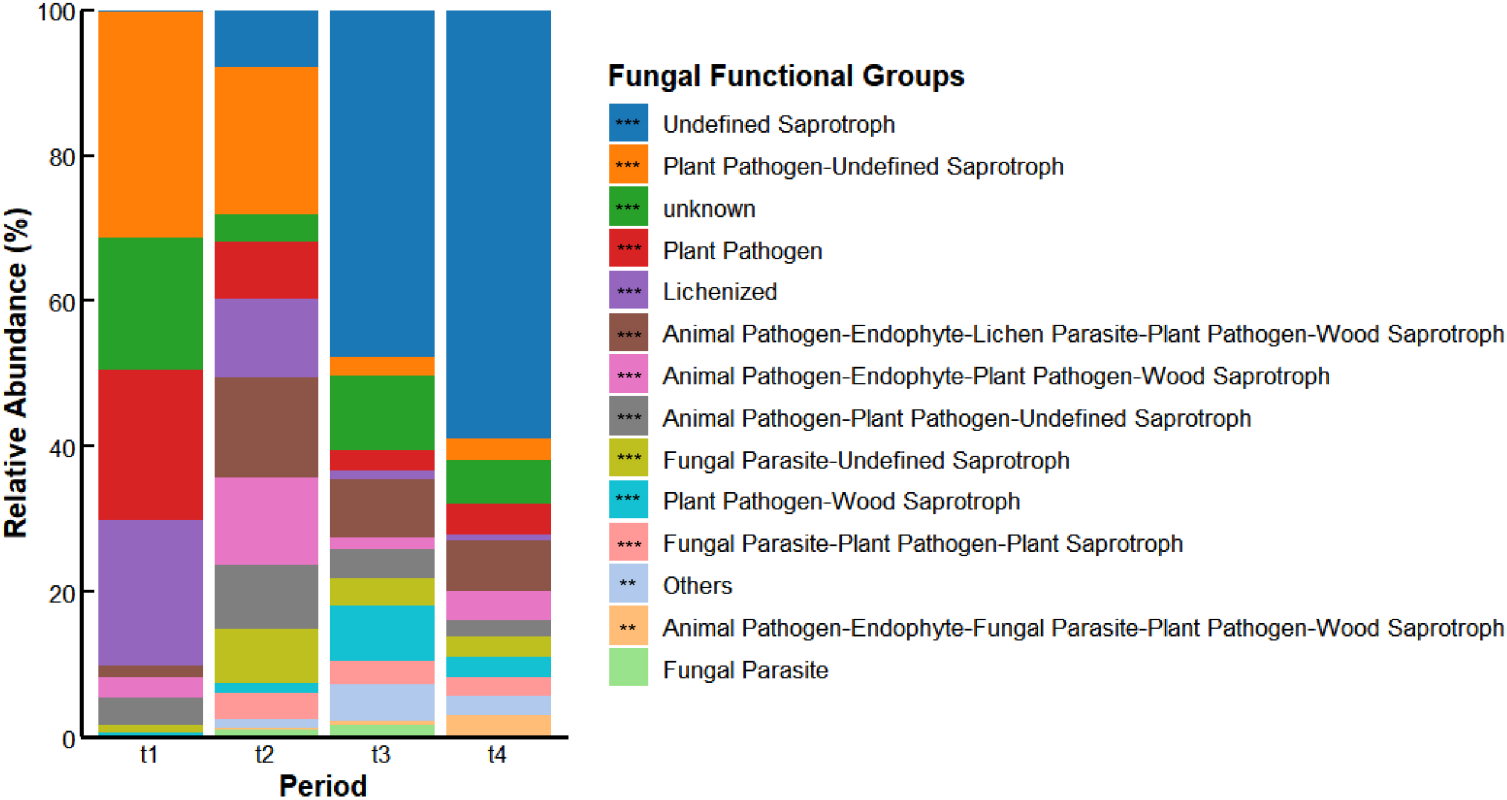
FUNGuild ecological functional prediction of litter fungal community in different stages.**p < 0.01, ***p < 0.001. t1: sampling in April 2022, t2: sampling in July 2022, t3: sampling in October 2022, t4: sampling in April 2023.

The FUNGuild results (**Figure 6**) indicated a significant variation in the relative abundance of fungal ecological functional guilds across distinct decomposition stages (*p* < 0.05). Specifically, Plant Pathogens–Undefined Saprotrophs, Plant Pathogens, and Lichenized exhibited notably higher relative abundances at t1 and t2 in comparison to t3 and t4. In contrast, the relative abundance of Undefined Saprotrophs exhibited a progressive rise over time, emerging as the predominant fungal ecological functional guild at t3 (47.68%) and t4 (58.96%).

### 3.6 Correlations between litter nutrients, structural components and fungal ecological function prediction

**Figure 7:**
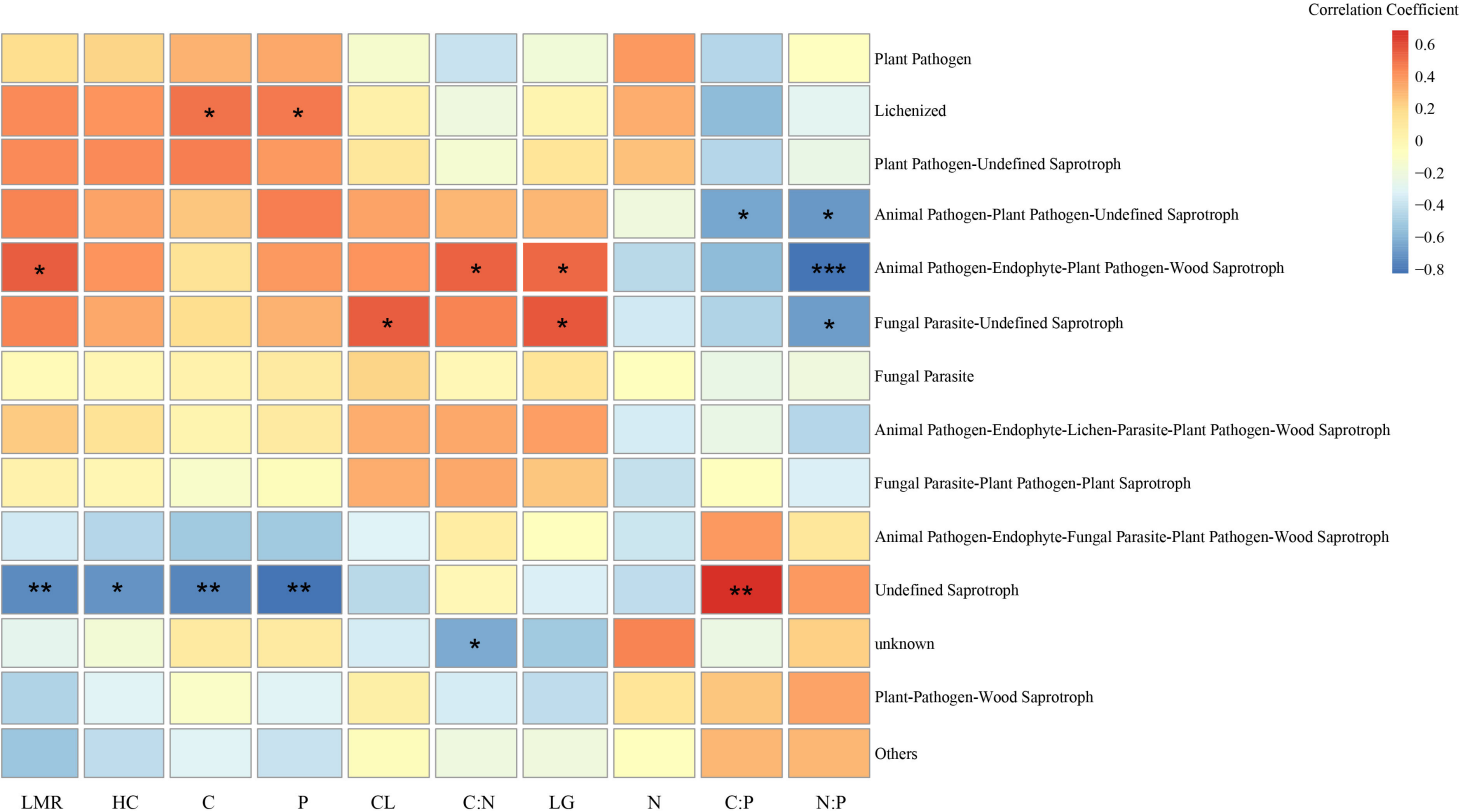
Relationship between fungal functional groups and nutrient element of litter. The color gradient on the right represents Pearson’s correlation coefficient values, ranging from -0.8 (dark blue, strong negative correlation) to 0.6 (dark red, strong positive correlation). *p < 0.05, **p < 0.01, ***p < 0.001. LMR (litter mass remaining), LG (lignin), CL (cellulose), HC (hemicellulose).

Correlation analysis of fungal ecological functional groups and nutrient elements in *Betula platyphylla* litter (**Figure 7**) revealed significant associations. Fungal Parasite-Undefined Saprotroph, Animal Pathogen-Endophyte-Lichen Parasite-Plant Pathogen-Wood Saprotroph showed negative correlations with N:P ratio and positive correlations with LG (*p* < 0.05). Animal Pathogen-Plant Pathogen-Undefined Saprotroph showed negative correlations with N: P ratio and C:P ratio (*p* < 0.05). Lichenized demonstrated positive correlations with C and P (*p* < 0.05). Conversely, Undefined Saprotroph displayed negative correlations with LMR, HC, C and P, while showing a positive correlation with C:P ratio (*p* < 0.05).

## 4 Discussion

### 4.1 Changes of nutrient elements and structural components during the decomposition of *Betula platyphylla* litter

Litter decomposition in forest ecosystems is a complex process governed by both intrinsic litter traits (e.g., species, chemical composition) and external environmental factors (e.g., temperature, precipitation) (Tang et al., 2014). In many ecosystems, such as temperate broadleaf and boreal coniferous forests, decomposition rates are typically slow, with the early stages often lasting 12 to 18 months (Bahnmann et al., 2018; Zhang et al., 2022). In our study site (WDLC), which is underlain by volcanic bedrock with coarse lava and gravel soils, litter decomposition is further constrained by low temperatures and surface drought (Huang et al., 2020). Consequently, after 18 months of decomposition, *Betula platyphylla* litter exhibited a mass loss of 38.27%, which aligns with the reported range for initial decomposition stages (litter mass remaining < 40%) (Heim and Frey, 2004).

During the early stages of decomposition, microorganisms utilize litter as a nutrient source, preferentially degrading readily available organic compounds such as sugars and fatty acids, which releases carbon (C) from the litter matrix (Cepáková and Frouz, 2015; Xue et al., 2016). Our study found that the C concentration in *Betula platyphylla* litter was significantly higher in the initial phases (t1, t2) than in the later phases (t3, t4), aligning with this rapid initial carbon release. If the N concentration of the litter is insufficient to meet microbial demand, microorganisms can immobilize additional N from the surrounding environment, leading to a net increase in litter N concentration (Berg and Staaf, 1981). This mechanism likely explains the initial increase in N concentration we observed from t1 to t2. The subsequent decrease from t3 to t4 may indicate that N reached a sufficiency threshold and began to be mineralized and released into the soil, thereby decreasing the litter N pool. Consequently, the litter C:N ratio exhibited a corresponding pattern, decreasing initially and then increasing. Microbial mineralization also drives P dynamics, converting organic P into plant-available phosphate and typically reducing the P concentration during litter decomposition (Richardson and Simpson, 2011; Olander and Vitousek, 2004). Furthermore, Manzoni proposed that net P release occurs when the litter C:P ratio is below a threshold of approximately 480 (Manzoni et al., 2010). The significant increase in the C:P ratio of *Betula platyphylla* litter (from 99.43 to 117.44) over the decomposition period in our study supports the notion that P release was occurring, consistent with the model’s prediction.

The degradation of structural components (cellulose, hemicellulose, and lignin) in *Betula platyphylla* litter exhibited distinct dynamics during decomposition. Consistent with previous reports (Ma et al., 2019), cellulose and hemicellulose were degraded more rapidly than lignin in our study. The primary reasons for this lag are the greater inherent recalcitrance of lignin, along with the lack of efficient lignin-degrading microbial communities in this environment, as well as the inhibition of relevant enzyme activities by mineral adsorption and the formation of recalcitrant lignin-mineral complexes (Hadland et al., 2024). In the early stages, microorganisms secrete enzymes such as cellulase and hemicellulase, which preferentially target the relatively simple polysaccharide structures of cellulose and hemicellulose, breaking them down into monosaccharides and other small organic molecules (Wang et al., 2020). In contrast, lignin, with its complex aromatic polymer structure, is more resistant to degradation. Its breakdown proceeds at a slower rate and is primarily mediated by specialized lignin-degrading enzymes (e.g., laccases and peroxidases) produced by certain fungal groups (Wang et al., 2020).

### 4.2 Changes of the fungal community during the decomposition of the *Betula platyphylla* litter

Fungi are key drivers of litter decomposition, secreting a suite of extracellular enzymes that degrade complex structural polymers such as cellulose, hemicellulose, and lignin (Purahong et al., 2014). Fungal communities typically exhibit more pronounced successional patterns than bacterial communities during decomposition (Zhan et al., 2021). In our study of *Betula platyphylla* litter in the volcanic lava habitat, Ascomycota and Basidiomycota were the predominant phyla, a finding consistent with reports from other nutrient-stressed environments (Yang, 2021; Chen et al., 2021; Ye et al., 2023). This suggests that environmental filtering by the volcanic substrate shapes the local fungal assemblage.

Ascomycota and Basidiomycota play complementary functional roles. Ascomycota, often dominant in early stages, are particularly adept at degrading labile carbon sources like cellulose, thereby facilitating rapid decomposition (Manici et al., 2024). Basidiomycota are crucial for breaking down more recalcitrant compounds, especially lignin, and fulfill diverse ecological roles including saprotrophy and symbiosis (Lundell et al., 2010; Manici et al., 2024). Their co-dominance in our system underscores their joint importance in driving litter decomposition and nutrient cycling within these volcanic forest ecosystems.

In volcanic lava, *Lophiostoma* and *Niesslia* were dominant fungal genera during the decomposition of *Betula platyphylla* litter. In various ecosystems, fungi of the genus *Lophiostoma* are recognized as key decomposers of plant litter, where they collaborate with other microorganisms to break down debris, thereby contributing to nutrient cycling and soil development (Bahnmann et al., 2018). Similarly, fungi belonging to the genus *Niesslia* are commonly reported as saprotrophs in natural environments. They are proficient in decomposing plant litter through the production of a diverse array of extracellular enzymes capable of degrading complex organic polymers such as cellulose and lignin, which facilitates the breakdown of plant residues and promotes nutrient cycling (Gams et al., 2019).

### 4.3 Relationship between nutrient elements and structural components of *Betula platyphylla* litter and fungal community

Fungal succession during litter decomposition is closely linked to changes in substrate chemistry. In the initial stages, readily available C and N provide essential energy and nutrients for microbial colonizers, supporting early fungal growth (Chen et al., 2019). Ascomycota, which dominated our early samples (t1, t2), are efficient degraders of hemicellulose and cellulose. Their activity releases C-rich compounds, contributing to the observed initial increase in the litter C:N ratio, which is inherently inversely related to N concentration (i.e., as N immobilizes or is released, the ratio changes). Furthermore, high initial concentrations of these polysaccharides likely provided a readily available resource base that promoted the high initial abundance and activity of Ascomycota.

In contrast, the later-stage increase in Basidiomycota abundance (peaking at t2) coincides with the decomposition of more recalcitrant lignin. Lignin degradation by Basidiomycota requires the production of nitrogen-rich oxidative enzymes (e.g., laccase, manganese peroxidase) (Wang et al., 2020). Therefore, sufficient N availability, potentially from the prior mineralization facilitated by early colonizers, may enhance the efficiency of this process. At the genus level, the temporal patterns of specific decomposers align with their known functions. For instance, the increased abundance of *Niesslia* over time corresponds with the gradual decline in cellulose, consistent with its reported cellulolytic capabilities (Gams et al., 2019). Similarly, the dominance of *Alternaria* at specific stages (e.g., t2) may relate to its role in nutrient liberation, which can influence soil fertility (Lou et al., 2013; Wang et al., 2023).

### 4.4 Changes in ecological functional groups of fungi during the decomposition of *Betula platyphylla* litter

The functional profiles of fungal communities in *Betula platyphylla* litter varied significantly across the four decomposition stages, indicating a shift in their ecological roles over time. Analysis of ecological functional guilds revealed a transition from a prevalence of Plant-Pathogenic fungi in the initial stages to a dominance of Saprotrophic fungi in the later stages. This successional pattern is commonly observed during litter decomposition, where specialized saprotrophs become increasingly important for breaking down recalcitrant organic matter (Floudas et al., 2012; Li et al., 2011). Saprotrophic fungi are particularly proficient at secreting a diverse suite of hydrolytic and oxidative extracellular enzymes, which are crucial for the degradation of complex polymers in litter, thereby driving nutrient release and cycling within the ecosystem.

The Undefined Saprotroph guild showed negative correlations with quantities of HC, C, and P, but a positive correlation with the C:P ratio in *Betula platyphylla* litter, suggesting a potential dominance of Undefined Saprotroph in the breakdown of recalcitrant compounds during advanced decomposition stages (Pan et al., 2023). The observed higher C:P ratio may indicate a preference for carbon substrate utilization under conditions of limited phosphorus availability, aligning with the metabolic strategy of saprotrophic fungi to enhance carbon utilization efficiency in nutrient-deficient environments (Pánek et al., 2024). Consistent with established understanding, fungi with saprotrophic and parasitic functions often employ nitrogen-intensive enzymatic pathways (e.g., for lignin degradation), which can lead to a reduction in the N:P ratio of the substrate (Boddy and Watkinson, 1995). Our observed decrease in the litter N:P ratio in later stages agrees with this mechanism. In contrast, Lichenized fungi showed positive correlations with C and P concentrations, possibly attributable to their symbiotic nature. Lichenized fungi, in partnership with photosynthetic algae or cyanobacteria, fix atmospheric CO_2_, while the fungal partner facilitates phosphorus acquisition from the substrate through extensive mycelial networks, jointly promoting the retention of both carbon and phosphorus (Pan et al., 2023).

## 5 Conclusions

This study elucidates the coupled dynamics of nutrient cycling and fungal community succession during the early decomposition of *Betula platyphylla* litter in the Wudalianchi volcanic lava habitat. We observed a significant decrease in C and P concentrations, while N displayed a trend of initial increase followed by decrease, leading to marked shifts in stoichiometric ratios (C:N, N:P, C:P). The structural components cellulose and hemicellulose were degraded more rapidly than lignin. Fungal community analysis revealed Ascomycota and Basidiomycota as the dominant phyla, with *Lophiostoma* and *Niesslia* as key decomposer genera. Their simultaneous presence may enhance the functional stability and resilience of nutrient cycling in these volcanic forest ecosystems. The succession of these communities was primarily driven by changes in litter mass remaining (LMR), C, and N. Furthermore, a clear functional transition occurred, from a prevalence of Plant-Pathogenic fungi in early stages to a dominance of Saprotrophic fungi in later stages. These findings underscore the critical and interconnected roles of specific fungal taxa and functional guilds in driving litter decomposition and nutrient cycling under the unique environmental constraints of volcanic ecosystems. A primary limitation of this work is the sample size, which may limit the statistical resolution of some analyses. To validate and extend our conclusions, future work should prioritize enhanced replication and longer-term monitoring.

## Data Availability

The original contributions presented in the study are publicly available. This data can be found here: NCBI SRA, accession PRJNA1425655.
